# Anticancer Potential of Quercetin on Oral Squamous Cell Carcinoma: A Scoping Review and Molecular Docking

**DOI:** 10.1055/s-0044-1789016

**Published:** 2024-09-30

**Authors:** Dhona Afriza, Utmi Arma, Raefany Faslah, Wastuti Hidayati Suriyah

**Affiliations:** 1 Department of Oral Biology, Faculty of Dentistry, Baiturrahmah University, Kota Padang, Sumatera Barat, Indonesia; 2 Department of Oral Medicine, Faculty of Dentistry, Baiturrahmah University, Kota Padang, Sumatera Barat, Indonesia; 3 Department of Oral Medicine, Faculty of Dentistry, Baiturrahmah University, Kota Padang, Sumatera Barat, Indonesia; 4 Department of Oral Biology, Faculty of Dentistry, University of YARSI, DKI, Jakarta, Indonesia

**Keywords:** quercetin, BCl-2, oral cancer, scoping review, molecular docking

## Abstract

Oral squamous cell carcinoma (OSCC) is a type of cancer that has a low survival rate and high recurrence and metastasis rates. To date, there is still no effective treatment for OSCC. Various types of cancer, including OSCC, have reported quercetin to act as an anticancer agent, but there is no clear research data on how it may affect OSCC. To determine the anticancer potential of quercetin in OSCC, we conducted a scoping review, and to determine the interaction of quercetin with one of the proteins that plays a role in carcinogenesis, namely, BCL-2, we conducted molecular docking. The scoping review process was conducted based on the Preferred Reporting Items for Systematic Reviews and Meta-Analyses Extension for Scoping Reviews. The scoping review was searched by collecting articles related to the research topic in Google Scholar, PubMed, ScienceDirect, Cochrane, and EBSCOhost databases. All of the literature records found during the search were imported into the Mendeley software to remove duplication. Nine studies were generated after the titles and abstracts were reviewed according to the inclusion and exclusion criteria. After the full-text screening, no studies were excluded, leaving nine publications determined to be eligible for inclusion in the scoping review. Quercetin showed effects on inhibiting cancer invasion, migration, proliferation, and many protein expressions, as well as increasing cell apoptosis. Molecular docking was done for quercetin and BCl-2 protein. Doxorubicin was utilized as a comparison ligand. The *in silico* study was utilized using AutoDock Vina, AutoDock Tools 1.5.6, Biovia Discovery Studio 2021, and PyMol. Molecular docking indicated quercetin has a strong binding affinity with BCl-2 protein (ΔG –7.2 kcal/mol). Both scoping review and molecular docking revealed that quercetin is a promising candidate for anticancer agent.

## Introduction

More than 90% of all oral cancers are oral squamous cell carcinomas (OSCCs), which are more common in men than in women. Low survival rates and high rates of recurrence, invasion, and metastasis are characteristics of OSCC. 1 2 3 Although many therapies have been applied for OSCC, the 5-year survival rate is still around 60%, due to therapy resistance and side effects. Obviously, OSCC requires a more potent treatment. 2 3 4

In the treatment of cancer, natural compounds should exhibit low harmful side effects, low cost, the absence of systemic toxicity, multitarget properties, and a certain ability to enhance the effects of conventional anticancer drugs. 5 6 The use of natural and/or synthetic drugs for chemoprophylaxis aims to inhibit, reverse, or suppress OSCC. Previous research suggests that foods rich in nutrients such as flavonoids, vitamin C, vitamin D, β-carotene, and curcumin can prevent the development of oral cancer. 7 8 Other natural compounds such as green tea extract, resveratrol, garcinol, anthocyanins, and other phenolics from berries, isothiocyanates, lycopene, genistein, thymoquinone, and Salvadora persica L. can also suppress OSCC. 9

A plant flavonol called quercetin is produced from flavonoid polyphenols, which are frequently present in foods like green tea, onions, grapes, apples, peppers, and vegetables. Many studies have reported that quercetin acts as an anticancer agent in various types of cancer, including OSCC. 10 There is no conclusive evidence on the effects of quercetin on OSCC, despite reports that it acts as an anticancer for a variety of cancers, including OSCC. This scoping review is intended to accurately assess the existing scientific evidence and determine the extent to which current research has investigated the potential of quercetin in OSCC, as well as to provide guidance for future lines of research to be undertaken to improve drug discovery for OSCC therapy, especially quercetin.

The prosurvival protein BCl-2 has a considerable impact on oncogenesis. Most cancer types have high levels of BCl-2 expression, which is associated with a poor prognosis due to its antiapoptotic impact. 1 The basal layers of a healthy epithelium express BCl-2. The early phases of epithelial carcinogenesis are hypothesized to be caused by the overexpression of the BCl-2 protein. BCl-2 overexpression modifies programmed cell death, causing cells that are unable to die to remain. 2 11 In this study, we also want to determine the interaction of quercetin with the BCL-2 protein by molecular docking.

## Methods

### Scoping Review

The scoping review protocol followed the Preferred Reporting Items for Systematic Reviews and Meta-Analyses Extension for Scoping Reviews (PRISMA-ScR) and also followed the PRISMA-ScR checklist. 12 This scoping review seeks to provide an answer to this main question: what is the empirical evidence for the anticancer potential of quercetin in OSCC?

### Search Strategy

A scoping review was conducted by searching publications from Google Scholar, PubMed, ScienceDirect, Cochrane, and EBSCOhost databases published between 2014 and January 2024. The determination of eligibility criteria and keywords in this scoping review was made based on the Population, Concept, and Context framework. 12 13

The search strategy was performed using keywords with the Medical Subject Headings terms: ((quercetin) OR (divertin) OR (3,3′,4',5,7-Pentahydroxyflavone)) AND ((Oral Squamous Cell Carcinoma) OR (Oral Cancer) OR (Oral Cavity Squamous Cell Carcinoma) OR (Oral Tongue Squamous Cell Carcinoma)). All of the literature records found during the search were imported into the Mendeley software to remove duplication.

### Eligibility Criteria

Titles and abstracts that might be relevant were screened for eligibility using the Mendeley software. The studies that were included had the following criteria: (1) studies related to the anticancer potential of quercetin in OSCC; (2) the population in this study were proteins that play a role in OSCC, OSCC cell lines, and experimental animals with OSCC; (3) articles created between 2014 and January 2024; (4) in English; (5) in the form of original papers; and (6) experimental ( *in silico* , *in vitro* , and *in vivo* ) studies; (7) intervention is only with quercetin, not in combination with other substances. The following criteria were used to exclude articles: (1) studies were systematic reviews and meta-analyses; (2) there was no time limit on the search; and (3) studies that did not examine the potency of quercetin on OSCC.

### Study Selection and Data Extraction

The eligibility criteria were applied independently by two authors (D.A. and R.F.). The selection process for the articles had two steps: first, screening titles and abstracts for articles that met the inclusion criteria; then, reading the full text of the selected articles; and then, excluding articles that did not meet the inclusion criteria. Next, extract data from selected articles. Data were collected based on the first author, year of publication, research methods, assessment, and conclusion of the results. The full texts of potentially eligible studies were subsequently screened independently by D.A., R.F., and U.A. This data was checked in several rounds. Any differences of opinion among the three authors were resolved through discussion with all authors until an agreement was reached. The details of the study selection process are documented in the PRISMA flow diagram.

### Critical Analysis and Evidence Synthesis

Quercetin has been reported to have anticancer potential, including in OSCC, but it is still unclear how quercetin may act as an anticancer agent in OSCC. This scoping review explores the current evidence regarding the anticancer activity of quercetin in OSCC, identifies gaps in the evidence, and can guide future research. The main results are presented in descriptive tables using a systematic method approach and are discussed in depth and critically.

### Molecular Docking Procedure

#### Programs and Software

This research method was experimental *in silico* with the molecular docking of quercetin with the protein BCl-2. AutoDock Vina, AutoDock Tools 1.5.6, Biovia Discovery Studio 2021, and PyMol were used in this investigation. The SDF file for the three-dimensional quercetin and doxorubicin ligands were acquired from PubChem. The BCl-2 protein's three-dimensional structure was retrieved from the Protein Data Bank (PDB) with the PDB code 4MAN. Doxorubicin was utilized as a comparison ligand.

#### Ligand and Protein Preparation

The chemical structure of the quercetin ligand in SDF file format was converted to PDB file format using Discovery Studio Biovia 2021. Then, the ligand structure in PDB form was changed to PDBQT form with AutoDock Tools 1.5.6 (ADT) so that it meets the requirements to run AutoDock Vina.

For virtual screening and docking validation using native ligand, native ligand in the BCl-2 protein (PDB: 4MAN) was isolated and stored as PDB files. By eliminating the atomic coordinates of the water molecules, including polar hydrogen, calculating Gasteiger charges for protein structures, and converting protein structures from PDB file format to PDBQT format, the BCl-2 protein structure was made ready for structure-based virtual screening and molecular docking procedures.

#### Validation of Protein-Ligand Complex Structures

The native ligands on the target protein should be redocked for docking validation. The native ligand was separated from the protein using Biovia Discovery Studio 2021. In this study, the native ligand C48 H52 Cl N7 O8 S was docked to the BCl-2 protein (PDB code: 4MAN). The expected binding-free energy (kcal/mol), which measures how tightly a ligand binds to the receptor, is calculated using the scoring system included in AutoDock Vina. A greater negative binding-free energy is a sign of stronger binding. The approach is considered legitimate if the root mean square deviation value generated is ≤ 2A when the candidate drug and target protein can be docked in the same grid box region. 14 15 16

#### Molecular Docking

The AutoDock Vina program has been used for molecular docking. The candidate ligand was docked with the protein, where the binding site corresponds to the size and center of the grid box, which has been obtained when redocking the native ligand with the BCl-2 protein (4MAN). Redocking of native ligands on proteins allowed protein binding sites to be obtained for this investigation. Notepad was used to create the setup file for AutoDock Vina.

### Analysis and Visualization

The docking conformation is determined based on the greatest affinity at the appropriate site. The results of the docking simulation were displayed using PyMol and Discovery Studio Biovia 2021 (DeLano Scientific LLC, United States).

## Results

### Scoping Review

**Fig. 1:**
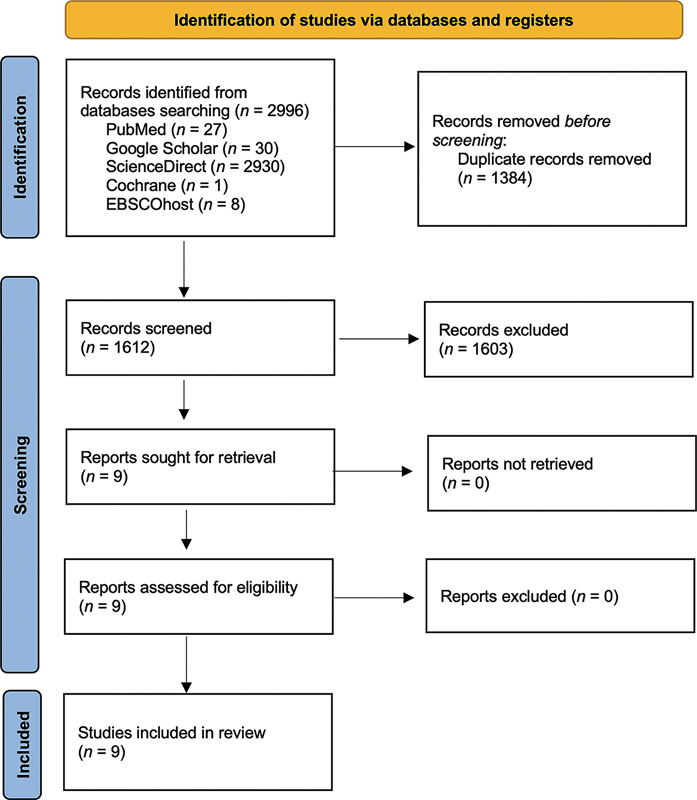
The Preferred Reporting Items for Systematic Reviews and Meta-Analyses (PRISMA) flow diagram shows the identification and selection process of the studies included in this coping review.

Fig. 1 provides a summary of the method used to choose the studies that would be included in the review. There are various steps to the publication selection procedure. The first stage is to search for publications through the Google Scholar, PubMed, ScienceDirect, Cochrane, and EBSCOhost databases. Based on the method of studies selection, 30 studies were obtained from Google Scholar, 2,930 ScienceDirect, 27 from PubMed, 1 Cochrane, and 8 EBSCOhost. A total of 2,996 studies will be screened by Mendeley. In the second stage, all articles were screened for duplication using Mendeley's application, resulting in 1,384 duplicate studies. The results of duplication screening left 1,612 studies. The third stage was screening by filtering articles by matching the inclusion and exclusion criteria for titles and abstracts. After screening titles and abstracts, a total of nine full-text studies were evaluated. There were no studies eliminated after reading the full texts. The scoping review eventually contained nine studies.

Table 1 provides a summary of the characteristics of the nine studies included in this scoping review. 10 17 18 19 20 21 22 23 24 All research is experimental. There are two sorts of research: *in vitro* and *in vivo* . The population in the study varied; hamsters and BALB/c nude mice were used in the *in vivo* study, and the other used cell lines (HSC-3, SCC-25, HGFs, Tca8113, HSC-6, SCC-9, OSC20, HN22, HaCaT, nHOK, CAL-27, YD10B, and YD38 cell lines). Various types of parameters are used to assess the potential of quercetin on OSCC, including: the examination of apoptosis, invasion, and migration; the examination of cell migration through protein levels of PI3K (phosphoinositide 3-kinases), nuclear factor kappa B (NF-κB), matrix metalloproteinase (MMP)-2, and MMP-9; the examination of apoptosis through messenger ribonucleic acid; NF-κB p50 and p65; Bcl-2 and Bax genes; the activities of caspase-3, caspase-8, and caspase-9; miR-22 expression; caspase-3 activity; and caspase 3/7 activity. Examination of proliferation and invasion was performed through levels of miR-1254 and CD36.

**Table 1:** Results of literature research

No.	References	Research methods	Assessment	Results conclusion
1.	Chan et al (2016) 17	*In vitro* on HSC-3, FaDu cell lines	Migration, invasion, colony growth, MMP-2, MMP-9	Quercetin efficiently inhibits the cellular migration and invasion of HSC-3 and FaDu cell lines. Quercetin significantly suppressed transcriptional activation MMP-2 and MMP-9 in both HSC-3 and FaDu cells
2.	Zhang et al (2017) 18	*In vivo* in hamsters	mRNA, p50 and p65, NF-κB, Bax, and Bcl-2 genes	In the DMBA-induced carcinogenesis hamster model, quercetin decreased tumor incidence and promoted apoptosis via increasing the expression of Bax, decreasing the expression of Bcl-2, and inhibiting NF-κB
3.	Ma et al (2018) 19	*In vitro* on SAS cell lines	Apoptosis, ROS, Ca ^2+^ , and caspase-3, caspase-8, and caspase-9 activities	In SAS cells, quercetin causes morphological changes in the cells, reduces cell viability, causes apoptosis, stimulates the generation of ROS and Ca ^2+^ , and boosts the activities of caspase-3, caspase-8, and caspase-9
4.	Zhang et al (2019) 20	*In vitro* on Tca8113 and SAS cell lines, *in* *vivo* in BALB/c nude mice	miR-22 level, WNT1/-catenin, and cell viability and apoptosis in Tca8113 and SAS cells. Tumor size every week	In OSCC cells, quercetin induces miR-22 expression and inhibits the WNT1/-catenin pathway, which decreases cell viability and promotes cell apoptosis. Quercetin inhibited OSCC tumor growth partly through regulating miR-22/WNT1/β-catenin axis *in vivo*
5.	Zhao et al (2019) 21	*In vitro* on SCC-9 and HSC-6 cell lines	Migration and invasion, cell viability, the concentrations of microRNA-16 (miR-16) and homeobox A10 (HOXA10), as well as their interactions, were examined	By controlling miR-16 and HOXA10 and reducing metalloproteinase-9 (MMP-9) and MMP-2 in oral cancer cells, quercetin prevents cell viability, migration, and invasion
6.	Kim et al (2020) 22	*In vitro* on OSC20, SAS, HN22, HaCaT, and nHOK cell lines	Cell viability, G2 phase cell cycle, migration potential, EMT and MMPs, EMT-TFs, TGF-β1	Quercetin accelerated the G2 phase of the cell cycle and decreased cell viability, suppressed the migration potential, and regulated EMT, MMPs, and EMT-TFs in OSCC cells
7.	Chen et al (2021) 23	*In vitro* on CAL-27 cell lines	Proliferation and invasion, levels of miR-1254 and CD36	By increasing miR-1254 and decreasing CD36, quercetin significantly reduced OSCC invasion and proliferation
8.	Huang et al (2022) 24	*In vitro* on SAS cell lines	Apoptosis, caspase-3 activity, caspase 3/7 activity	In human tongue SCC SAS cells, quercetin causes apoptosis and mitochondrial damage via the JNK, ERK1/2, and GSK3 −/− signaling pathways
9.	Son and Kim (2023) 10	*Invitro* on YD10B and YD38 cell lines	Cell cycle arrest and apoptosis	Quercetin repressed cell proliferation with G1 cell cycle arrest and induced apoptosis signals in both cells

Abbreviations: DMBA, 7,12-dimethylbenz[a]anthracene; EMT, epithelial-mesenchymal transition; MMP, metalloproteinase; mRNA, messenger ribonucleic acid; NF-κB, nuclear factor kappa B; OSCC, oral squamous cell carcinoma; ROS, reactive oxygen species; TGF-β1, transforming growth factor-β1; TFs, transcription factors.

Table 2 displays the quality appraisal's findings. A detailed description of the guidelines utilized for assigning points to each category may be found in the Supplementary Appendix (available in the online version). All studies can provide information related to the context of this study about the anticancer potential of quercetin.

**Table 2:** Summary of critical appraisal for each study (based on the Critical Appraisal Skills Program [CASP])

No.	Study	Validity of the study design	Study method	Result	Utility of the findings locally	Appraisal summary
1.	Chan et al, 2016	Yes	Yes	Yes	Yes	Yes
2.	Zhang et al, 2017	Yes	Yes	Yes	Yes	Yes
3.	Ma et.al, 2015	Yes	Yes	Yes	Yes	Yes
4.	Zhang et al, 2019	Yes	Yes	Yes	Yes	Yes
5.	Zhao et al, 2019	Yes	Yes	Yes	Yes	Yes
6.	Kim et al, 2020	Yes	Yes	Yes	Yes	Yes
7.	Chen et al, 2021	Yes	Yes	Yes	Yes	Yes
8.	Huang et al, 2022	Yes	Yes	Yes	Yes	Yes
9.	Son and Kim, 2023	Yes	Yes	Yes	Yes	Yes

### Molecular Docking

#### Docking Validation

**Fig. 2:**
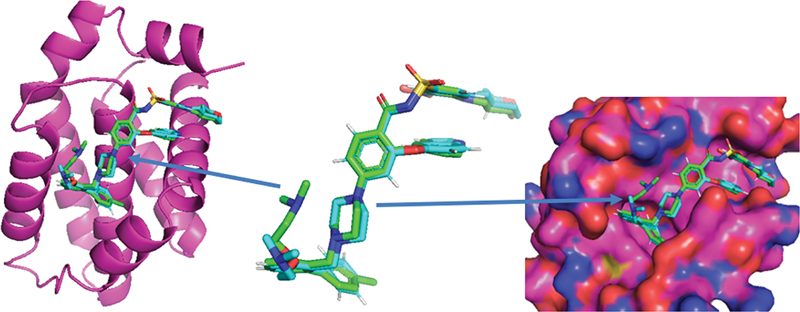
Redocking native ligand. The redocking crystal structure in the best position is shown in green, and the conformational ligand crystal structure is blue.

To validate the results, the native ligand redocking to the BCl-2 protein (PDB code: 4MAN) has been carried out. The result of redocking was a grid box with center *x* = –11.917, *y* = 7.972, and *z* = 9.11, and size *x* = 24, *y* = 50, and *z* = 26. This redocking also results in a binding affinity of –11.1 kcal/mol. The results of the validation are presented in Fig. 2 .

**Fig. 3:**
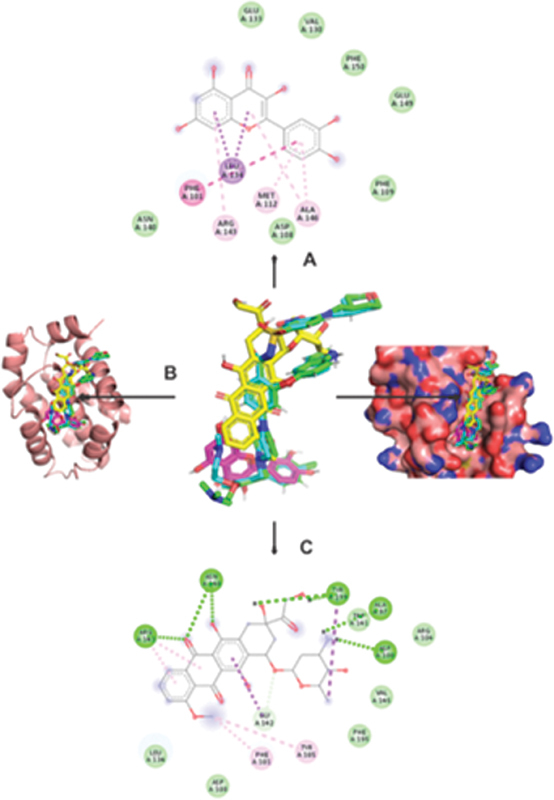
The best conformation model and binding interactions of the ligands, quercetin ( **A** ), doxorubicin ( **C** ), and superimposition of all ligands at the best binding site position ( **B** ) on the BCl-2 protein.

The amino acid residues and binding affinities for the quercetin ligands' interactions with the BCL-2 receptor were determined by the molecular docking. The results of molecular docking between quercetin and BCl-2 are displayed in Table 3 and Fig. 3 .

**Table 3:** The importance of binding affinities and the role of amino acid residues in the interactions between quercetin and doxorubicin compounds' ligands and the BCl-2 receptor at their optimal binding site positions

Ligands	Binding free energy ΔG (kcal/mol)	Amino acids involved and distance (Â)		
		Hydrogen binding interaction	Hydrophobic interaction	Electrostatic interaction
Quercetin	−7.2	–	Leu134 (3.29), Leu134 (3.65), Phe101 (5.2), Ala146 (5.44), Arg143 (5.29), Met112 (4.82), Ala146 (4.8)	–
Doxorubicin	−7.7	Tyr199 (2.22), Tyr199 (2.57), Ala97 (3.04), Asp100 (2.1), Asn140 (3.18), Asn140 (2.8), Arg143 (3.1), Gly142 (3.71)	Tyr199 (3.5), Gly142 (3.88), Arg143 (4.97), Arg143 (5.08), Phe101 (5.42), Tyr105 (4.51)	–

Note: The binding affinity of the investigated ligands shows particular interactions with the residues of amino acid around the receptor site.

## Discussion

Numerous studies in recent years have shown that natural substances and their bioactive compounds have anticancer properties, known as phytochemicals, in the treatment of oral cancer. Quercetin is a phytochemical that has been considered a signal transduction pathway modulator to prevent, inhibit, or reverse carcinogenesis. Numerous studies have demonstrated the significant role quercetin and its metabolites play in preventing cancer and oxidative stress. These compounds are found in a variety of plants. Quercetin is also widely used as an antioxidant and anti-inflammatory. Quercetin is utilized to cure a wide range of illnesses, including coronary heart disease, diabetes, and cancer, according to several research. 25 26 In OSCC, quercetin has been recognized as a possible antitumor agent. In this work, we investigated the function of quercetin in the treatment of OSCC in more detail, through a scoping review and molecular docking.

The results of the scoping review found that quercetin may act as an antitumor agent for OSCC *in vitro* and *in vivo* in several ways, namely, by inhibiting cell growth and invasion/migration, reducing tumor incidence, inducing apoptosis, suppressing metastatic ability, and suppressing the progression of OSCC. *In vitro* studies were performed with various OSCC cell lines, while one *in vivo* study was performed on hamsters. 10 17 18 19 20 21 22 23 24

Quercetin inhibited migration and invasion by suppressing the expression of MMP-2 and MMP-9 in the HSC-3 cells. 17 Quercetin prevents cell viability, migration, and invasion by controlling miR-16 and HOXA10 and reducing MMP-9 and MMP-2 in oral cancer cells. 21 Quercetin inhibited the propensity for migration, sped up the G2 phase of the cell cycle, reduced cell viability, and controlled epithelial-mesenchymal transition (EMT), MMPs, and EMT-transcription factors in OSCC cells. 22 Quercetin induces apoptosis by increasing the expression of miR-22, deactivating the WNT1/-catenin pathway, 20 and upregulating caspase-3, caspase-8, and caspase-9 activity. 19 In OSCC cells, quercetin suppresses metastatic ability by decreasing the expression level of transforming growth factor-β1-induced EMT and MMP significantly. 22 According to Chen et al, 23 quercetin significantly suppresses invasion and cell proliferation by activating the miR-1254/CD36 signaling pathway in OSCC cells. A study by Huang et al 24 stated that in human tongue SCC SAS cells, quercetin induces apoptosis and mitochondrial damage through the JNK, ERK1/2, and GSK3-α/β signaling pathways. Quercetin induced apoptosis by activating the p38 signaling pathway and inhibited cell proliferation with G1 cell cycle arrest in YD10B and YD38 cell lines. 10

In another study conducted by Singh et al, 27 combining quercetin and resveratrol was able to suppress growth and increase death in Cal-33 cells. Quercetin and cisplatin can inhibit the activation of NF-kB, which is an antiapoptotic protein in human OSCC (Tca-8113 and SCC-15 cell lines). 28

Zhang et al's study on hamsters was the sole *in vivo* investigation performed. 18 The study revealed that, by increasing Bax expression, decreasing BCl-2 expression, and preventing transactivation of the NF-κB gene involved in oncogenesis, quercetin reduces tumor incidence and promotes apoptosis. 19 Zhang et al also conducted research on BALB/c nude mice, stating that quercetin inhibited OSCC tumor growth partly through regulating the miR-22/WNT1/β-catenin axis *in vivo* . Li et al 20 suggested that the combination of quercetin and cisplatin could inhibit the growth of xenograft tumors in a mouse model. In animal models, quercetin has the potential to suppress the development of OSCC, either alone or in combination with other drugs.

The results of the scoping review showed that quercetin has potential as an anticancer for OSCC in various ways, namely: inhibits cell growth, inhibits invasion and migration of cells, reduces tumor incidence, induces apoptosis or cell death, inhibits cell viability, and suppresses the progression of OSCC. Most of the study was performed *in vitro* with various cell lines and also looked at various effects on apoptotic and antiapoptotic proteins. Although the study was conducted on various types of oral cancer cells, all the results showed that quercetin was able to suppress OSCC activity. However, every cell has unique characteristics. Different cell types will have different growth characteristics, as will cancer cells; the growth rate varies throughout various tumor types. 29

Cell lines are often used instead of primary cells to study biological processes. Because cell lines are genetically modified, they do not always accurately replicate primary cells. This may change its response to stimuli, phenotypic, and original function. Cell lines are also frequently contaminated by HeLa cells and mycoplasma during distribution. Therefore, cell lines may not adequately represent primary cells and may provide different results. 30 It would be better if further research investigates the effects of quercetin on primary cells. More future research should be performed *in vivo* and clinical trials.

It is important to take into account a few of this scoping review's shortcomings. First, there are limitations in collecting articles through databases. A literature search was conducted in the five major databases: Google Scholar, ScienceDirect, PubMed, Cochrane, and EBSCOhost. Therefore, additional relevant studies might have been missed. Second, the limitations of reading the full text can result in the risk of bias, such as selection bias, insufficient blinding, and selective outcome reporting.

Zhang et al 18 stated that quercetin can reduce BCl-2 expression in OSCC. The development of numerous cancers, including OSCC, is linked to the antiapoptotic protein BCl-2. 2 31 Apoptosis is the most significant mechanism of cell death in response to malignancy therapy and is mostly regulated by BCl-2. Targeting the BCl-2 protein may improve apoptosis with chemotherapeutic agents. 31

Molecular docking has been performed between quercetin and the BCl-2 protein. According to the results obtained, quercetin can bind strongly with BCl-2, producing a binding-free energy (binding affinity) of –7.2 kcal/mol. There are seven amino acid residues (Leu134 (3.29 Å), Leu134 (3.65 Å), Phe101 (5.2 Å), Ala146 (5.44 Å), Arg143 (5.29 Å), Met112 (4.82 Å), and Ala146 (4.8)) produced in hydrophobic interactions, but no amino acids are produced in hydrogen and electrostatic interactions.

Doxorubicin as a comparison ligand produces a binding-free energy of –7.7 kcal/mol, greater than quercetin. The amino acid residues produced in the hydrophobic interaction are 6 amino acids (Tyr199 (3.5 Å), Gly142 (3.88 Å), Arg143 (4.97 Å), Arg143 (5.08 Å), Phe101 (5.42 Å), Tyr105 (4.51 Å)), 8 amino acids (Tyr199 (2.22 Å), Tyr199 (2.57 Å), Ala97 (3.04 Å), Asp100 (2.1 Å), Asn140 (3.18 Å), Asn140 (2.8 Å), Arg143 (3.1 Å), Gly142 (3.71 Å)) generated in hydrogen interaction, while no amino acid residues are generated in electrostatic interactions. Compared with the interaction between quercetin and BCl-2, the interaction between doxorubicin and BCl-2 produces greater free energy and more amino acids, so doxorubicin has a stronger bond with BCl-2.

The main goal of drug development is to discover small molecules that bind strongly to specific biomacromolecules. Almost all biological processes depend on protein-ligand binding. Particular biological recognition is determined by underlying physical and chemical interactions at the molecular level. Finding a chemical ligand that binds to a particular target protein and either inhibits or stimulates it via ligand binding is a crucial step in the drug development process. In the early phases of drug development, identifying a ligand that binds a specific protein with high affinity is a significant problem. 32

Binding free energy, often known as binding affinity, is the single most important early indicator of drug efficacy and is used to determine the affinity of biomolecular interactions and drug efficacy. 33 34 The strength of the interaction between the ligand and the protein is measured by the magnitude of the binding affinity, which is frequently directly proportional to the potential of the ligand. 33 The more negative the value, the better the bond-free energy between the protein and the ligand. 35

Hydrophobic interactions predominated and made a significant contribution, even though polar and hydrogen-bonding interactions aid in the right orientation of the compound (or its functional groups) to achieve the maximal interaction. The degree of affinity between the medication and the receptor is determined by the total strengths of these connections. 36 It is generally accepted that the main thermodynamic force behind the binding of small molecule ligands to their associated protein receptors is a result of hydrophobic interactions. There have been several empirical scoring systems created to calculate protein-ligand binding affinities, and they are all dominated by measures of the hydrophobic contact between the protein and the ligand. 37

According to Lipinski's criteria, ligands (inhibitors) are considered to have the potential to enter the cell membrane and be absorbed by the body if they meet the following criteria: (1) molecular weight < 500 g/mol; (2) the number of hydrogen bonding proton donor groups < 5; (3) the number of hydrogen bond proton acceptor groups < 10; and (4) the logarithmic value of the partition coefficient in water and 1-octanol < 5 (C log P). 38 39 Based on Lipinski's criteria, nordentatin has high bioavailability for the body because it fulfills all of Lipinski's criteria. Doxorubicin does not meet Lipinski's criteria because it has a molecular weight > 500 g/mol, namely, 543.52 g/mol, so it has poor bioavailability for the body. Donor count for hydrogen bonds > 5 (6), number of hydrogen bond acceptors > 10 (12).

Doxorubicin has been used to treat oral cancer. But the effectiveness of doxorubicin therapy is limited by drug resistance mechanisms, including resistance to OSCC. Doxorubicin also has adverse side effects, especially cardiotoxicity and myelosuppression. Reactive oxygen species produced by doxorubicin have been the most studied cause of cardiotoxicity and are believed to act as major triggers of several forms of cell death, including apoptosis, necrosis, and autophagy. 40 41 42 43

It has been acknowledged that BCl-2 is a potential target for the creation of brand new antitumor medications. 31 The overexpression of the BCl-2 protein is linked to the early stages of epithelial carcinogenesis. Several studies have shown that BCl-2 expression is high in poorly differentiated carcinomas. 2 31 Increased expression of BCl-2 is not only important for oral carcinogenesis but also influences disease progression as it increases the survival rate of neoplastic cells, allows new genetic mutations to occur, and gives them higher resistance to chemotherapy and radiotherapy. 44 BCl-2 is a protein whose overexpression and phosphorylation may be related to the regulation of cell growth, cell cycle, proliferation, deoxyribonucleic acid repair, and tumorigenesis. 31 According to the molecular docking results, quercetin can act as a BCl-2 inhibitor.

Molecular docking has limitations in explaining interactions between proteins and ligands due to the rigidity of proteins. For this reason, it is necessary to carry out further research using molecular dynamics and other experiments.

## Conclusion

In summary, our scoping review provides evidence that quercetin could potentially suppress OSCC, both *in vitro* and *in vivo* . Based on the molecular docking that has been performed, it also shows that quercetin has a strong binding affinity with BCl-2, thus showing that quercetin has the potential to be an anticancer. This scoping review and molecular docking are quite interesting and informative and can open up insights for future research. Both scoping review and molecular docking revealed that quercetin is a promising candidate for anticancer agent.
