# Supplementary material for: Anticancer Potential of Quercetin on Oral Squamous Cell Carcinoma: A Scoping Review and Molecular Docking
Source: Eur J Dent. 2024 Sep 30;19(1):15–23. doi: 10.1055/s-0044-1789016 (PMC11750326; doi:10.1055/s-0044-1789016)
Supplement: Supplementary file 1 — Supplementary Material [file 10-1055-s-0044-1789016-s2413294.pdf]

Supplementary Appendix

CASP Randomized Controlled Trial Standard Checklist

A. Section A: Is the basic study design valid for a randomized controlled trial?
1. Did the study address a clearly focused research question?
2. Was the assignment of participants to interventions randomized?
3. Were all participants who entered the study accounted for at its conclusion?

B. Section B: Was the study methodologically sound?
4 - Were the participants 'blind' to intervention they were given?
- Were the investigators 'blind' to the intervention they were giving to participants?
- Were the people assessing/analyzing outcome/s 'blinded'?
5. Were the study groups similar at the start of the randomized controlled trial?
6. Apart from the experimental intervention, did each study group receive the same level of care (that is, were they treated equally)?

C. Section C: What are the results?
7. Were the effects of intervention reported comprehensively?
8. Was the precision of the estimate of the intervention or treatment effect reported?
9. Do the benefits of the experimental intervention outweigh the harms and costs?

D. Section D: Will the results help locally?
10. Can the results be applied to your local population/in your context?
11. Would the experimental intervention provide greater value to the people in your care than any of the existing interventions?

Y: Yes
N: No
CT: Can't tell

No.	Study	A	B	C	D	Appraisal Summary
1.	Chan et al, 2016	1. Y<br>2. Y<br>3. Y	4. CT<br>5. Y<br>6. Y	7. Y<br>8. Y<br>9. CT	10. Y<br>11. CT	Y
2.	Zhang et al, 2017	1. Y<br>2. Y<br>3. Y	4. Y<br>5. Y<br>6. Y	7. Y<br>8. Y<br>9. CT	10. Y<br>11. CT	Y
3.	Ma et al, 2018	1. Y<br>2. Y<br>3. Y	4. Y<br>5. Y<br>6. Y	7. Y<br>8. Y<br>9.CT	10. Y<br>11. CT	Y
4.	Zhang et al, 2019	1. Y<br>2. Y<br>3. Y	4. CT<br>5. Y<br>6. Y	7. Y<br>8. Y<br>9.CT	10. Y<br>11. CT	Y
5.	Zhao et al, 2019	1. Y<br>2. Y<br>3. Y	4. CT<br>5. Y<br>6. Y	7. Y<br>8. Y<br>9.CT	10. Y<br>11. CT	Y
6.	Kim et al, 2020	1. Y<br>2. Y<br>3. Y	4. CT<br>5. Y<br>6. Y	7. Y<br>8. Y<br>9.Y	10. Y<br>11. CT	Y
7.	Chen et al, 2021	1. Y<br>2. Y<br>3. Y	4. Y<br>5. Y<br>6. Y	7. Y<br>8. Y<br>9.CT	10. Y<br>11. CT	Y
8.	Huang et al, 2022	1. Y<br>2. Y<br>3. Y	4. CT<br>5. Y<br>6. Y	7. Y<br>8. Y<br>9.CT	10. Y<br>11. CT	Y
9.	Son and Kim, 2023	1. Y<br>2. Y<br>3. Y	4. CT<br>5. Y<br>6. Y	7. Y<br>8. Y<br>9.CT	10. Y<br>11. CT	Y

 © 2024. The Author(s).
